# Down Regulation of Wnt Signaling Mitigates Hypoxia-Induced Chemoresistance in Human Osteosarcoma Cells

**DOI:** 10.1371/journal.pone.0111431

**Published:** 2014-10-27

**Authors:** Donald J. Scholten, Christine M. Timmer, Jacqueline D. Peacock, Dominic W. Pelle, Bart O. Williams, Matthew R. Steensma

**Affiliations:** 1 Michigan State University College of Human Medicine, Grand Rapids, Michigan, United States of America; 2 Van Andel Research Institute, Grand Rapids, Michigan, United States of America; 3 Helen DeVos Childen's Hospital, Spectrum Health System, Grand Rapids, Michigan, United States of America; University of Kentucky, United States of America

## Abstract

Osteosarcoma (OS) is the most common type of solid bone cancer and remains the second leading cause of cancer-related death for children and young adults. Hypoxia is an element intrinsic to most solid-tumor microenvironments, including that of OS, and is associated with resistance to therapy, poor survival, and a malignant phenotype. Cells respond to hypoxia through alterations in gene expression, mediated most notably through the hypoxia-inducible factor (HIF) class of transcription factors. Here we investigate hypoxia-induced changes in the Wnt/β-catenin signaling pathway, a key signaling cascade involved in OS pathogenesis. We show that hypoxia results in increased expression and signaling activation of HIF proteins in human osteosarcoma cells. Wnt/β-catenin signaling is down-regulated by hypoxia in human OS cells, as demonstrated by decreased active β-catenin protein levels and axin2 mRNA expression (p<0.05). This down-regulation appears to rely on both HIF-independent and HIF-dependent mechanisms, with HIF-1α standing out as an important regulator. Finally, we show that hypoxia results in resistance of human OS cells to doxorubicin-mediated toxicity (6–13 fold increase, p<0.01). These hypoxic OS cells can be sensitized to doxorubicin treatment by further inhibition of the Wnt/β-catenin signaling pathway (p<0.05). These data support the conclusion that Wnt/β-catenin signaling is down-regulated in human OS cells under hypoxia and that this signaling alteration may represent a viable target to combat chemoresistant OS subpopulations in a hypoxic niche.

## Introduction

Osteosarcoma (OS) is the most common type of solid bone cancer, mainly arising in children and young adults, and it remains the second leading cause of cancer-related death in this age group [1]. Osteosarcomas are aggressive, high-grade tumors, with about 20% of patients presenting with metastases [1]. OS is a genomically unstable and heterogeneous tumor, and therapeutic options have not improved over the past four decades [2], [3].

Hypoxia is intrinsic to most solid-tumor microenvironments and is associated with resistance to therapy, poor survival, and a malignant phenotype [4]. Cells respond to hypoxia through alterations in gene expression, mediated most notably through the hypoxia-inducible factor (HIF) class of transcription factors [5]. HIF itself is a heterodimeric transcription factor comprising an oxygen-sensitive α-subunit and a constitutively expressed β-subunit [6]. Under normoxia, prolyl hydroxylases hydroxylate the HIFα subunit using O_2_ as a substrate, and the hydroxylated HIFα can interact with the von Hippel-Lindau protein to ubiquitinate it and target it for degradation [7], [8]. Under hypoxic conditions, the lack of O_2_ prevents this hydroxylation, and heterodimerized HIF enters the nucleus to bind to hypoxia response elements and function as a transcription factor by interacting with other coactivators such as CBP/p300 [7]. Hypoxic regions have been identified in human osteosarcomas, and increased levels of HIF-1α are correlated with decreased disease-free survival and overall survival, as well as increased microvessel density and surgical stage [9], [10]. In OS cell lines, hypoxia increases cell migration and promotes chemoresistance independent of HIF-1α [11], [12]. Although signaling responses governed by HIFs have been studied extensively in different cancers, little is known about the contributing mechanisms of hypoxia-associated therapy resistance in OS and whether or not they are related to HIFs.

The Wnt/β-catenin signaling pathway contributes to the pathogenesis of numerous diseases including OS [13], [14], [15], [16], [17]. In the absence of Wnts, β-catenin undergoes phosphorylation at key serine and threonine residues via the “destruction complex”, which includes adenomatous polyposis coli (APC), axin2, casein kinase 1 (CK1), and glycogen synthase kinase 3 (GSK3). This targets β-catenin for subsequent ubiquitinylation and proteasomal degradation. Binding of Wnts to the Frizzled family of receptors and the LDL-receptor-related proteins (LRP) 5 and 6 co-receptors serves to inhibit the destruction complex, allowing β-catenin to enhance TCF/LEF-mediated transcription [14]. Wnt/β-catenin signaling activation contributes to chemoresistance in OS cells, and increased β-catenin expression has been correlated with shorter cumulative survival in OS patients [18], [19].

Although studies in colorectal cancer cells have demonstrated hypoxic interactions between HIFs and β-catenin, results are conflicting in terms of defining Wnt/β-catenin signaling alterations in response to hypoxia in osteoblasts [20], [21], [22]. Furthermore, no studies have examined whether Wnt/β-catenin signaling is involved in the cellular adaptation to hypoxia in OS. Here we show that that hypoxia results in increased expression and signaling activation of HIF proteins in human osteosarcoma cells. We also show that Wnt/β-catenin signaling is down-regulated under hypoxic conditions, and this down-regulation appears to receive contributions that are both HIF-independent and HIF-dependent. Finally, we show that hypoxia results in robust resistance of OS cells to doxorubicin treatment, which is part of the mainstay chemotherapy regimen for OS patients. This hypoxia-induced chemotherapy resistance can be reduced by further inhibition of the Wnt/β-catenin signaling pathway, suggesting a role for Wnt signaling antagonism in targeting hypoxic OS cell subpopulations.

## Materials and Methods

### General cell culture and hypoxia chamber

Human osteosarcoma cells (143B, MNNG/HOS, MG-63) were obtained from the ATCC (Manassas, VA). The human OS cell line 206-2 was derived from a consenting OS patient according to protocols approved by the local ethics committee (IRB full name: Spectrum Health Institutional Review Board; part of the Spectrum Health Research Protection Program). Written informed consent was obtained from human research subjects under an IRB-approved (IRB full name: Spectrum Health Institutional Review Board), musculoskeletal tumor and tissue acquisition protocol at Spectrum Health (2011-002). The 143B cells were cultured in Dulbecco's Modified Eagle Medium (DMEM; Gibco) supplemented with 1% L-glutamine, 1% penicillin/streptomycin, and 10% fetal calf serum (FCS); MNNG/HOS cells in Minimum Essential Medium with Earle's Salts (EMEM) supplemented with 1% L-glutamine, 1% penicillin/streptomycin, and 10% FCS; MG-63 cells in EMEM supplemented with 1% L-glutamine, 1% penicillin/streptomycin, 1% nonessential amino acids, 1% sodium pyruvate, and 10% FCS; and 206-2 cells in Minimum Essential Medium (MEM-α) supplemented with 1% L-glutamine, 1% penicillin/streptomycin, and 10% FCS. These cells were cultured under standard incubation conditions (37°C, 5% CO_2_) and were subcultured every 3–4 days. Incubation under hypoxic conditions was in a 856-HYPO model hypoxia chamber (PLAS Labs, Lansing, MI) set to 0.5% O_2_, 5% CO_2_, and 37°C.

### Silencing of HIF-1α and HIF-2α

Bacterial glycerol stocks containing the shRNA plasmid of interest were purchased from Sigma Aldrich. shRNA sequences used were as follows: HIF-1α; TRCN0000003810, TRCN0000003811; HIF-2α; TRCN0000003804, TRCN0000003806. Lentiviral particles were produced in the 293FT packaging cell line using the ViraPower Lentiviral Packaging Mix (Invitrogen) according to the manufacturer's guidelines. Lentiviral infection of MNNG/HOS cells was done in the presence of 8 µg/mL polybrene (Millipore) according to the manufacturer's guidelines before puromycin selection (2 µg/mL). A non-targeting shRNA was used as a control (Sigma Aldrich; SHC002V).

### Quantitative reverse transcription PCR (qrt-PCR)

Total RNA was extracted from OS cells using TRIzol reagent (Invitrogen). Complementary DNA synthesis was performed using 500 ng RNA according to the instructions with the High Capacity cDNA Reverse Transcription Kit (Invitrogen). 18S primers (F:CCGCAGCTAGGAATAATGGA; R:CGGTCCAAGAATTTCACCTC), axin2 primers (F:AAGCAAGCGATGAGTTTGCCTGTG; R:ACAGCCAAGACAGTTCACAAGAGC), and c-myc primers (published previously) were obtained from Integrated DNA Technologies (Coralville, IA) [23]. Quantitative polymerase chain reactions were performed using SYBR Select Mastermix (Applied Biosystems, Carlsbad, CA) in 10 µL reactions. Polymerase chain reaction was performed according to the manufacturer's instructions using a StepOnePlus cycler (Applied Biosystems). Data were analyzed using the 2^−ΔΔC^_T_ method [24]. Data were presented as mean and standard deviation using Microsoft Excel, with statistical significance being determined using a two-tailed, one-sample *t*-test with µ = 1 using R and significance set at α = 0.05 [25].

### Western blot analysis

Whole-cell lysates were washed with PBS and scraped on ice in lysis buffer (RIPA lysis buffer) supplemented with complete Protease Inhibitor Cocktail (Roche). Protein concentration was measured using the BCA assay (Pierce), and 20 µg of whole-cell lysate was run on a 10% SDS/polyacrylamide gel. The proteins were transferred onto a nitrocellulose membrane, and membranes were probed overnight at 4°C with the appropriate primary antibody; antibodies used were as follows: active β-catenin, β-catenin, Oct4 (Cell Signaling Technologies); HIF-1α (R&D Systems), HIF-2α (Abcam), β-tubulin (Santa Cruz), and actin (Millipore). Membranes were then probed with a horseradish-peroxidase-conjugated secondary antibody for 1.5 hours at room temperature before detection using an enhanced chemiluminescence (ECL) detection system (Pierce). Images shown are representative of 2 independent experiments. Western blot densitometry was performed using ImageJ software (Rasband, W.S., ImageJ, U. S. National Institutes of Health, Bethesda, Maryland, USA, http://imagej.nih.gov/ij/, 1997–2014).

### Chemoresistance assay

Cells were plated into 96-well white-walled plates (Greiner Bio-One) at 1×10^3^ cells/well and allowed to adhere for 24 hours before drug treatments. The cells were then exposed to serial dilutions of doxorubicin (0–10 µM, LC Labs) and were placed in either normoxic or hypoxic conditions (as described above). After an additional 72 hours, cell viability was determined using the CellTiter-Glo Luminescent Cell Viability assay (Promega). Data were normalized to an untreated control well and graphed, and half maximal inhibitory concentration (IC_50_) values were calculated from the dose-response curve as the concentration of doxorubicin that produced a 50% decrease in the mean luminescence relative to untreated control wells. Statistical tests (two-tailed paired *t*-test and two-tailed two-sample *t*-test) were performed using R with significance set at α = 0.05. The tankyrase inhibitor XAV939 (Santa Cruz) and porcupine inhibitor IWP-2 (Sigma Aldrich) were used at a 10 µM concentration, with dimethyl sulfoxide (DMSO; Fischer) alone used as a control.

## Results

### HIFs are present and can be induced via hypoxia in human OS cells

**Figure 1:**
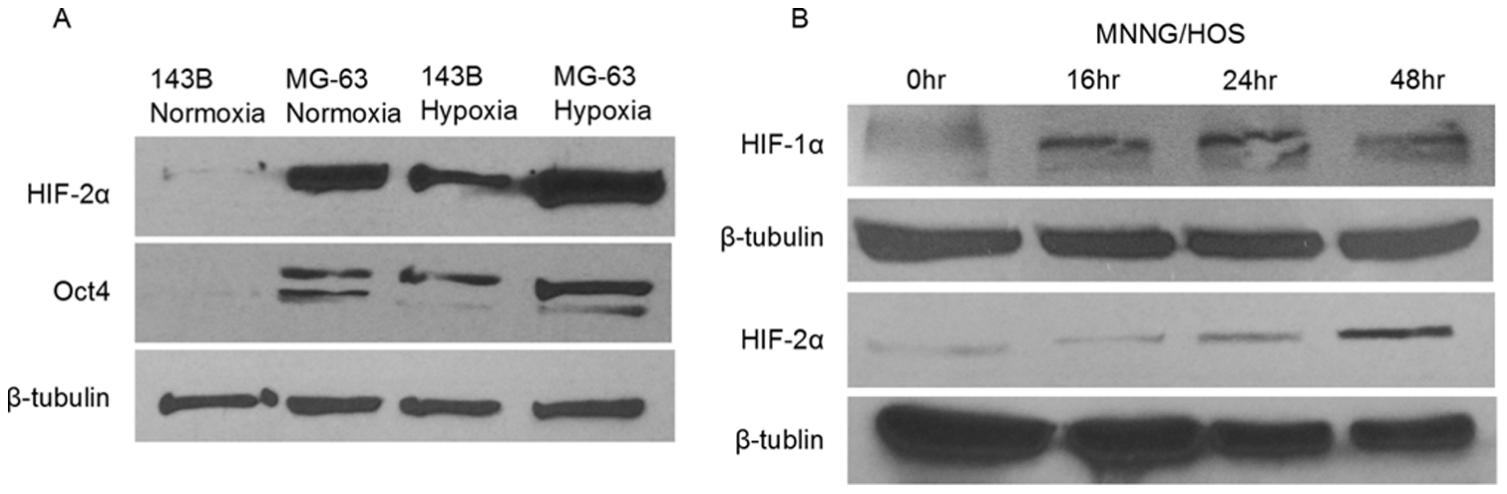
HIFs are present and active in human OS cells. A, 143B and MG-63 human OS cells showed increases in HIF-2α protein expression by western blot when cultured for 72 hour under hypoxic conditions (0.5% O_2_). The HIF-2α downstream target, Oct4, was also increased, indicating HIF signaling activity. β-tubulin was the loading control. B, MNNG/HOS human OS cells were cultured under hypoxia for up to 48 hour and compared to a normoxia (0 hour) control. Both HIF-1α and HIF-2α levels were increased as seen by western blot. β-tubulin was used as a loading control.

The goal of this study was to identify targetable signaling changes taking place under hypoxic conditions related (or unrelated) to the HIF transcription factors in human OS cells. We first needed to validate the presence of HIFs and examine their activity and induction under normoxic and hypoxic conditions. We cultured human OS cell lines (143B, MG-63, MNNG/HOS) under hypoxic (0.5% O_2_) or normoxic conditions for 72 hours and examined the protein levels of HIF-1α, HIF-2α, and a downstream target of HIF-2α, Oct4, by western blot. The expression of HIF-2α and Oct4 in the MG-63 and 143B cell lines increased under the hypoxic conditions relative to normoxic conditions (Figure 1A). When we examined HIF protein expression at shorter time points in the MNNG/HOS cell line, we found that both HIF-1α and HIF-2α were induced by culturing cells under hypoxic conditions for increasing time periods (Figure 1B). Results of two independent replicates were quantified, showcasing the increase in HIF protein levels (Figure S1). These results show that hypoxia stimulates both the expression and signaling activity of HIF proteins in human OS cells.

### Wnt/β-catenin signaling is down-regulated in response to hypoxia

**Figure 2:**
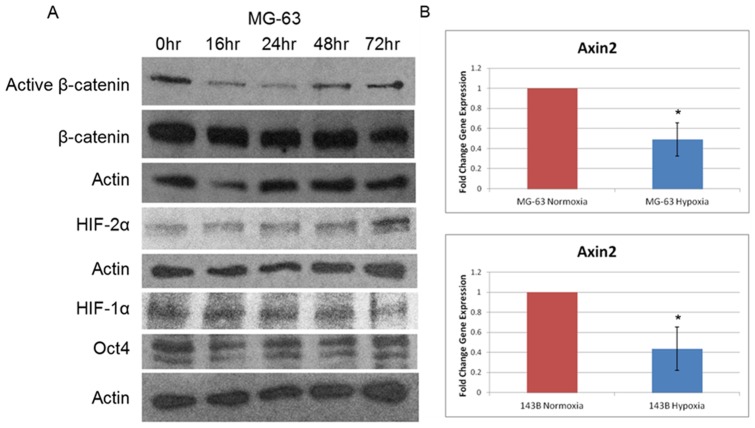
Hypoxia down-regulates Wnt/β-catenin signaling in human OS cells. A, MG-63 human OS cells were cultured under hypoxia over 72 hour, and increased HIF protein expression was observed by western blot. Protein levels of active β-catenin were decreased under hypoxic conditions relative to normoxic conditions (0 hour). Actin was the loading control. B, Axin2 mRNA levels were determined as a measure of Wnt/β-catenin signaling under hypoxic conditions (72 hour, 0.5% O_2_) via quantitative reverse-transcription PCR (qrt-PCR) normalized to normoxia. Asterisks indicate statistical significance (*p<0.05).

Previous reports have described interactions between β-catenin and HIF-1α, thereby linking the Wnt signaling pathway to the hypoxic response [22]. Furthermore, there have been conflicting reports on the role of changes in Wnt/β-catenin signaling in response to hypoxia in osteoblasts [20], [21]. We set out to characterize alterations to Wnt/β-catenin signaling in response to hypoxia in human OS cells. In MG-63 cells, we examined protein expression for different durations of hypoxia via western blots and again noticed increased HIF-1α and HIF-2α expression (Figure 2A). We also noticed decreased protein levels of active β-catenin throughout the hypoxic time course, suggesting that Wnt/β-catenin signaling was down-regulated (Figure 2A). This finding was supported through western blot quantification of active β-catenin protein levels (Figure S2). This active (hypophosphorylated) β-catenin antibody recognizes the stabilized form of β-catenin that has not been phosphorylated by GSK-3β, which thereby is active in cell-cell adhesion and canonical Wnt signaling. Levels of active β-catenin strongly correlate with its transcriptional activity [26]. As an alternative measure of Wnt/β-catenin signaling activity, we assessed axin2 mRNA expression using qrt-PCR. Axin2 is a well-established target of β-catenin-dependent transcription, and its mRNA expression is routinely used as a marker of Wnt/β-catenin signaling [27]. After 72 hours of hypoxia, axin2 mRNA was reduced to 49% in the MG-63 cell line and 44% in the 143B cell line relative to their respective normoxic controls (Figure 2B, p<0.05). These results using active β-catenin protein levels and axin2 mRNA expression indicate that Wnt/β-catenin signaling activity is decreased under hypoxic conditions in human OS cells.

### Hypoxic Wnt/β-catenin signaling alterations show evidence of both HIF-dependent and HIF-independent mechanisms

**Figure 3:**
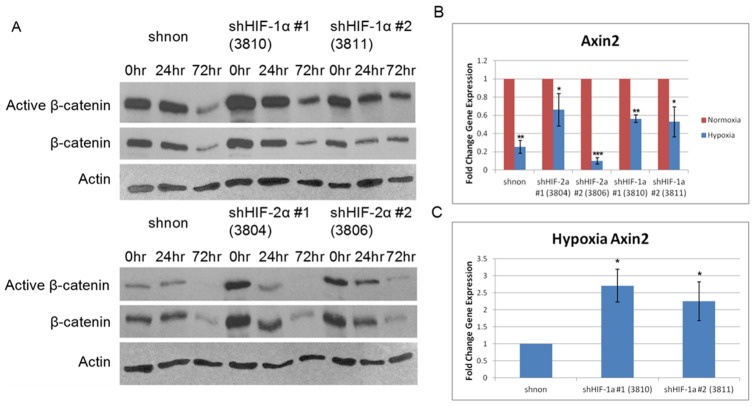
Wnt/β-catenin signaling down-regulation is both dependent and independent of HIF expression. A, Levels of active β-catenin were determined by western blot at different hypoxia time points for each of the shRNA in MNNG/HOS cells. Overall levels of active β-catenin decreased regardless of HIF expression, although the magnitude of decrease was not equal across all lines. Active and total β-catenin protein levels were decreased at the 72 hour time point. Actin was the loading control. B, Decreased Wnt/β-catenin signaling activity under hypoxia was confirmed by measuring axin2 mRNA levels via qrt-PCR in MNNG/HOS cells. Hypoxia (72 hour, 0.5% O_2_) resulted in decreased axin2 mRNA expression relative to normoxia. C, When analyzed relative to the shnon hypoxia mRNA, axin2 mRNA was increased in the shHIF-1α MNNG/HOS cell lines. Asterisks indicate statistical significance (*p<0.05, **p<0.01, ***p<0.001). shNON: non-targeting shRNA; HIF shRNAs used are indicated in parentheses.

The observed down-regulation in Wnt/β-catenin signaling raises mechanistic questions, especially regarding the potential involvement of HIF proteins. To determine whether the expression of either HIF protein is needed for the decrease in Wnt/β-catenin signaling, we stably transduced MNNG/HOS cells with shRNA targeting either HIF-1α or HIF-2α. We confirmed knockdown at the mRNA (80–90% knockdown) and protein level specific to each HIF protein relative to a non-targeting shRNA control for two independent shRNA's targeting each HIFα subunit (data not shown). Next we examined Wnt/β-catenin signaling activity under hypoxic conditions between the shHIF and non-targeting shRNA MNNG/HOS cells. Active β-catenin protein levels were decreased under hypoxic conditions in both the non-targeting shRNA and the shHIFα cell lines (Figure 3A), as was seen before in the MG-63 cell line (see Fig. 2A). There was also a decrease in total β-catenin at the 72 hour time point (Figure 3A). We assessed axin2 mRNA expression across the shHIF-modified cell lines, comparing normoxic to hypoxic conditions to determine whether β-catenin transcriptional activity was truly altered, or whether the decrease in active β-catenin protein only reflected a decrease in total β-catenin. There was a statistically significant decrease in axin2 mRNA expression under hypoxic conditions in the non-targeting shRNA MNNG/HOS cell line (Figure 3B, p<0.01), just as noted earlier for the MG-63 and 143B lines (see Figure 2B). That decrease was also consistent in the shHIF-1α- and shHIF-2α-treated MNNG/HOS cell lines, indicating that HIF-independent mechanisms contribute to Wnt/β-catenin signaling down-regulation under hypoxia (Figure 3B, p<0.05). However, when the data were normalized to the hypoxia shnon control, axin2 mRNA expression in both hypoxia shHIF-1α cell lines was increased, implying that HIF-1α contributes to Wnt/β-catenin signaling down-regulation (Figure 3C, p<0.05). Thus, this data suggests that both HIF-dependent and HIF-independent mechanisms contribute to the hypoxia-mediated down-regulation of Wnt/β-catenin signaling in human OS cells.

### Hypoxia results in chemoresistance of human OS cells to doxorubicin

**Figure 4:**
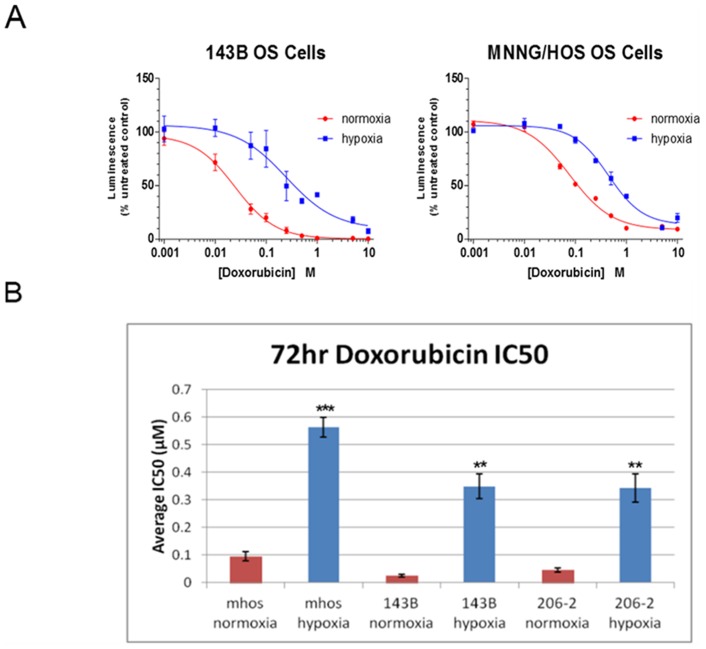
Hypoxia results in chemoresistance of human OS cells to doxorubicin. A, Dose-response curves for the 143B and MNNG/HOS (mHOS) cell lines treated with increasing concentrations of doxorubicin under normoxic and hypoxic conditions (72 hour, 0.5% O_2_). Luminescence (viability) was determined as a percent of untreated control (0 µM doxorubicin). B, Average half maximal inhibitory concentration (IC_50_) values were obtained from the dose-response curves and compared between normoxic and hypoxic conditions for the cell lines MNNG/HOS (mHOS), 143B, and a patient-derived OS cell line, 206-2. Asterisks indicate statistical significance (**p<0.01, ***p<0.001).

Hypoxia has been shown to promote resistance to cytotoxic drugs in other human OS cell lines [12]. Thus we asked whether a more broad panel of OS cell lines, and particularly a patient-derived cell line isolate, show resistance under hypoxia to doxorubicin treatment, which is part of the mainstay chemotherapy regimen for OS patients [28]. We cultured both MNNG/HOS cells and 143B cells under normoxic and hypoxic conditions for 72 hours in the presence of increasing concentrations of doxorubicin. In both cell lines we saw a dramatic right shift in the dose–response curve under hypoxic conditions, indicating that these cells were more resistant to doxorubicin-mediated growth inhibition (Figure 4A). We calculated the average half maximal inhibitory concentration (IC_50_) between normoxic and hypoxic conditions for each cell line and found that hypoxia resulted in a statistically significant increase in the doxorubicin IC_50_ (MNNG/HOS, 5.9-fold increase; 143B, 13.6-fold increase, Figure 4B, p<0.01). We tested a patient-derived OS cell line (206-2) and found that it too was significantly more resistant to doxorubicin treatment under hypoxic conditions (7.5-fold increase, Figure 4B, p<0.01). We also examined the response to doxorubicin treatment under hypoxia and normoxia in MNNG/HOS cells in the context of either HIF-1 or HIF-2 knockdown. We were not able to determine any statistically significant difference between the non-targeting shRNA and the HIFα shRNA cell lines (Figure S3).

### Wnt/β-catenin signaling inhibitors sensitize hypoxic human OS cells to doxorubicin

**Figure 5:**
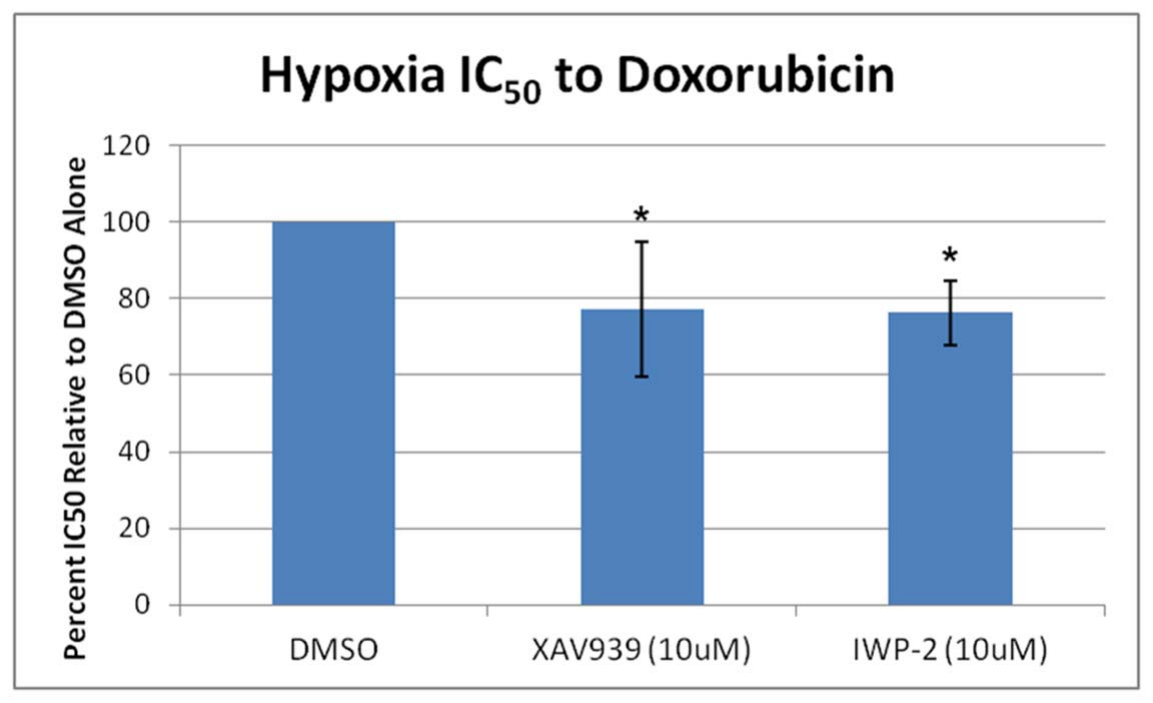
Further Wnt/β-catenin signaling inhibition sensitizes hypoxic OS cells to doxorubicin. MG-63 OS cells were treated with increasing concentrations of doxorubicin under hypoxic conditions in the presence of the tankyrase inhibitor XAV939, the porcupine inhibitor IWP-2, or DMSO alone. Half maximal inhibitory concentrations (IC_50_) were calculated, and the percent IC_50_ relative to DMSO alone was determined. Asterisks indicate statistical significance (*p<0.05).

Coupled with the findings of hypoxic Wnt/β-catenin down-regulation and the hypoxia-induced chemoresistance, we asked whether survival in the face of chemotherapy for a hypoxic OS cell depended on a threshold of Wnt/β-catenin signaling activity, and whether further inhibition of this pathway would sensitize the cell to chemotherapy. We chose to attenuate Wnt/β-catenin signaling under hypoxic conditions using the tankyrase inhibitor XAV939 [29]. XAV939 works by stabilizing axin2 and preventing its tankyrase-mediated degradation, thereby promoting the degradation of β-catenin and decreasing Wnt/β-catenin-dependent gene expression [29], [30]. We first validated that treatment with XAV939 results in decreased protein levels of active β-catenin and decreased mRNA expression of Wnt target genes axin2 and c-myc in MG-63 cells (Figure S4). We then treated MG-63 human OS cells under hypoxic conditions with 10 µM XAV939 (or DMSO alone as a control); treatment with XAV393 resulted in a 23% reduction in the 72-hour doxorubicin IC_50_ (Figure 5, p<0.05).

As another approach, we antagonized Wnt secretion using the porcupine inhibitor IWP-2 [31]. Porcupine is a member of the membrane-bound O-acyltransferase family of enzymes that is needed to add a palmitoyl group to Wnts. Such addition is required for Wnt secretion, and the inhibition of porcupine results in decreased Wnt secretion [31]. Treatment of MG-63 cells with IWP-2 resulted in decreased protein levels of active β-catenin and decreased mRNA expression of Wnt target genes axin2 and c-myc (Figure S4). Subsequently, treatment of MG-63 cells under hypoxic conditions with 10 µM IWP-2 resulted in a reduction in the 72-hour doxorubicin IC_50_ similar to that with XAV393 (24%; Figure 5, p<0.05). These data suggest that inhibition of Wnt/β-catenin signaling may serve as a target in effectively sensitizing hypoxic OS cell subpopulations to chemotherapy.

## Discussion

Tumor hypoxia and HIF expression are found in a wide range of solid tumors and are often associated with a poor prognosis [8]. Hypoxia can drive many processes that contribute to cancer progression, including altered metabolism, angiogenesis, pH regulation, and cell proliferation [32]. Many of these responses are mediated through the HIF class of transcription factors, because both HIF-1α and HIF-2α can influence the expression of key target genes for tumor growth and progression. It is no surprise that clinical data highlights the importance of increases in HIF on parameters of poor outcome in OS patients [9], [10]. So far, inhibition strategies targeting the HIF proteins directly have suffered from a lack of specificity [33]. It may be a more viable option to target the key downstream mediators of the hypoxic response. *VEGFA*, a well-characterized HIF target gene, is associated with poor prognosis in OS patients [34]. A single clinical trial is ongoing targeting VEGFA with bevacizumab in osteosarcoma (NCT00667342), however it is unclear whether this therapy is effective [35]. Downstream hypoxia/HIF targets have shown promise in other cancers, including VEGFA in various cancers and GLUT1 in renal cell carcinoma [36], [37], [38].

Although signaling mediated by HIFs constitutes a potent hypoxia response mechanism, there are many instances of hypoxia responses that are considered HIF-independent. Crucial signaling pathways other than HIFs, such as RAS and PI3K/mTOR, can function in hypoxia-induced angiogenesis [39]. These pathways can be activated under hypoxia through inhibition of the suppressive activity of PTEN and MAPK phosphatases via reactive oxygen species (ROS) [40], [41], [42]. Alternative signaling pathways and ROS mediators can in turn regulate HIF expression and activity, adding further complexity to the response to hypoxia [43], [44]. An understanding of crucial, downstream, HIF-independent signaling pathways that are regulated by hypoxia could lead to new therapeutic targets.

In this study, we tested the hypothesis that Wnt/β-catenin signaling is altered under hypoxic conditions in human OS cells, and we sought contributions of HIFs to such alteration. Other studies in osteoblasts, using exogenous HIF-1α and siRNAs targeting HIF-1α under normoxic conditions, have noted that HIF-1α inhibits Wnt signaling, but we used physiologic hypoxia-induced HIF-1α or HIF-2α in the context of their knockdown to examine Wnt signaling differences [45]. Through our work we were able to conclude Wnt/β-catenin signaling is down-regulated under hypoxia in OS cells, and that both HIF-independent and HIF-dependent mechanisms contribute to this finding, with HIF-1α standing out as an important mediator. Although the baseline β-catenin levels and the magnitude of its subsequent alteration under hypoxia differ among the cell lines used (MG-63 in Figure 2A, MNNG/HOS in Figure 3A), the general trend of β-catenin and canonical Wnt signaling decrease remained consistent. It may be that this baseline results from differences intrinsic to the origin and behavior of the cell lines themselves [46]. HIF-independent mechanisms explaining our findings could involve the Siah-1-p53 axis; Siah-1 increases under hypoxia in other cancer cell lines and results in the degradation of β-catenin dependent on p53 [47]. Studies in osteoblasts have specifically focused on HIF-1α driving a decrease in Wnt/β-catenin signaling through activation of sclerostin [20]. Future work will be needed to identify whether these or additional mechanisms are responsible for hypoxic Wnt/β-catenin signaling down-regulation in OS cells. It is also possible that since 100% knockdown of HIF expression was not achieved using RNA interference, a low level of HIF activity could be sufficient to govern canonical Wnt signaling changes, or that redundancy between HIF-1 and HIF-2 could result in compensation. This is an area of current ongoing investigation.

We also examined the chemoresistance of OS cells under hypoxic conditions, and assessed the efficacy of Wnt signaling inhibitors on sensitizing hypoxic OS cells to chemotherapy. Our results show that across multiple OS cell lines, including a patient-derived clinical sample, hypoxia promotes robust resistance to doxorubicin, and hypoxic OS cells can be sensitized to doxorubicin by simultaneously antagonizing the Wnt signaling pathway. This is not to say that hypoxia-mediated chemoresistance in OS cells is driven by down-regulated Wnt signaling per se, but instead that homeostasis of hypoxic OS cells may be susceptible to a second hit of Wnt signaling antagonism. Similar strategies for targeting a signaling pathway from multiple directions have proven effective in other cancers, as one study highlights that mTORC and HDAC inhibition mechanistically converged on the PI3K/AKT/mTOR pathway to reduce breast cancer cell viability [48]. Future directions would include determining if any synergism exists between doxorubicin and Wnt signaling antagonism. Others have shown that hypoxia promotes cell cycle arrest, which could be a contributing mechanism for hypoxia-induced chemoresistance [49]. We chose to normalize our chemoresistance assays to an untreated control under either normoxia or hypoxia to focus more closely on signaling pathway inhibition rather than proliferation differences. Future work examining hypoxia-induced proliferation differences and the signaling pathways responsible in OS will be important.

XAV939 and IWP-2 differ in their mechanisms of Wnt signaling inhibition, with XAV939 targeting Wnt signaling at the β-catenin level and IWP-2 targeting Wnt signaling at the Wnt secretion and subsequent membrane receptor level. A likely explanation for their similar effects on sensitizing hypoxic OS cells to chemotherapy is that this finding may depend on the presence of Wnts for β-catenin signaling. It will be important to identify which Wnt ligands are important for the hypoxic response, and their downstream effects on canonical and non-canonical Wnt signaling. Additionally, more potent and specific inhibitors of the Wnt signaling pathway than the ones used in this study are being used currently in clinical trials (NCT01351103). Our work serves as an *in-vitro* model demonstration of how cells under oxygen tensions similar to those in the tumor microenvironment may be susceptible to Wnt signaling antagonism. More work will need to be done to examine the biological efficacy of more potent and specific Wnt signaling inhibitors on hypoxic OS cells.

The clinical importance of our findings is clear, as a range of oxygen tensions (0–5% O_2_) can be observed within a solid tumor [50], [51], [52]. By targeting mechanisms that allow osteosarcoma cells to adapt to hypoxia within the solid tumor microenvironment, it is conceivable that the emerging pipeline of Wnt-targeted therapies can be leveraged to increase the effectiveness of current osteosarcoma drug regimens. Osteosarcoma is a very heterogeneous cancer, and data regarding the role of Wnt signaling in osteosarcoma is conflicting [2]. A recent genomic analysis of 46 early stage osteosarcoma specimens confirmed deletion of key genes in the canonical Wnt signaling pathway [53]. In this study and others, β-catenin activation has not been readily demonstrable by immunohistochemistry in human specimens [54]. Other studies confirm that osteosarcoma is responsive to Wnt signaling inputs, both within the canonical and non-canonical pathways [17], [55]. Using a systems biology approach, Wnt signaling pathways were identified as common mechanisms in human metastatic osteosarcoma models, suggesting a role for Wnt signaling antagonism in advanced stage OS [56]. Our experimental approach utilized metastatic cell lines and a chemoresistant patient-derived cell isolate to model advanced stage disease, and thus may be an important setting to investigate the use of Wnt-targeted therapies. Despite the complexity of signaling pathway alterations under hypoxia, whether related or unrelated to HIFs, the Wnt signaling pathway may serve as a viable target for hypoxia-induced chemoresistant OS cell subpopulations.

## Supporting Information

Figure S1 **Quantification of HIF protein levels under hypoxia.** Western blots from two independent experiments culturing MNNG/HOS cells under hypoxia for different time periods were quantified using ImageJ software. Band intensity is shown relative to β-tubulin.
(TIF)

Figure S2 **Quantification of active β-catenin protein levels under hypoxia.** Western blots from two independent experiments culturing MG-63 cells under hypoxia for different time periods were quantified using ImageJ software. Band intensity is shown relative to actin.
(TIF)

Figure S3 **Effects of HIF knockdown on OS cell resistance to doxorubicin.** 72 hour doxorubicin IC_50_ values are shown for two independent shRNA's targeting either HIF-1α or HIF-2α in MNNG/HOS cells. No significant difference was noted under hypoxic conditions (n = 3).
(TIF)

Figure S4 **Validation of Wnt/β-catenin Signaling Inhibition by XAV939 and IWP-2.** MG-63 cells were treated with either 10 µM XAV939 or IWP-2, with DMSO serving as a control. Active β-catenin protein levels were decreased by both XAV939 and IWP-2 compared to DMSO, and XAV939 treatment resulted in decreased in total β-catenin levels as determined by western blot. Treatment with XAV939 and IWP-2 also resulted in decreased mRNA expression of Wnt target genes axin2 and c-myc.
(TIF)
