# Supplementary figures and images for: Down Regulation of Wnt Signaling Mitigates Hypoxia-Induced Chemoresistance in Human Osteosarcoma Cells
Source: PLoS One. 2014 Oct 27;9(10):e111431. doi: 10.1371/journal.pone.0111431 (PMC4210185; doi:10.1371/journal.pone.0111431)

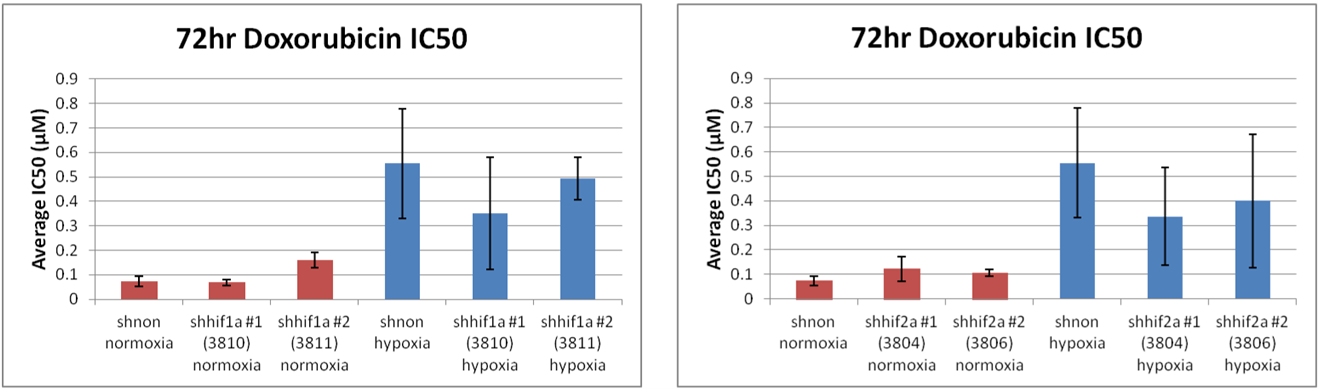

Supplement: Figure S3 — Effects of HIF knockdown on OS cell resistance to doxorubicin. 72 hour doxorubicin IC50 values are shown for two independent shRNA's targeting either HIF-1α or HIF-2α in MNNG/HOS cells. No significant difference was noted under hypoxic conditions (n = 3). (TIF) [file pone.0111431.s003.tif]
